# Properties and Characterization of Sunflower Seeds from Different Varieties of Edible and Oil Sunflower Seeds

**DOI:** 10.3390/foods13081188

**Published:** 2024-04-13

**Authors:** Zhenyuan Li, Fei Xiang, Xuegang Huang, Manzhu Liang, Sarina Ma, Karim Gafurov, Fengying Gu, Qin Guo, Qiang Wang

**Affiliations:** 1 Institute of Food Science and Technology, Chinese Academy of Agricultural Sciences, Key Laboratory of Agro-Products Processing, Ministry of Agriculture and Rural Affairs, Beijing 100193, China; li_zhenyuan@163.com (Z.L.); 82101221137@caas.cn (F.X.); huangxuegang2022@163.com (X.H.); lmz9610@163.com (M.L.); masarina@163.com (S.M.); gufengying@caas.cn (F.G.); wangqiang06@caas.cn (Q.W.); 2 Bukhara Engineering and Technological Institute, Bukhara 200100, Uzbekistan; kgafurov@yahoo.com

**Keywords:** sunflower seed varieties, raw material quality, physical and chemical indicators, processing characteristics

## Abstract

Sunflower seeds, oil, and protein powder are rich in nutritional value, but the quality of different varieties of sunflower seeds is quite different, and the comprehensive comparative analysis characteristics of edible and oil sunflower seeds are still unclear. The comprehensive analysis and comparison of the raw material indicators, physicochemical properties, and processing characteristics of four edible and four oil sunflower seed varieties were investigated. The results showed that the engineering properties, texture characteristics, single-cell structure, and oil, protein, and starch granule distribution were different between edible and oil sunflower seeds. The composition of fatty acids and amino acids was different among edible, oil sunflower seeds and different varieties. The oleic acid (18.72~79.30%) and linoleic acid (10.11~51.72%) were the main fatty acids in sunflower seed oil, and in amino acid composition, the highest content was glutamic acid (8.88~11.86 g/100 g), followed by aspartic acid (3.92~4.86 g/100 g) and arginine (4.03~4.80 g/100 g). Sunflower meal proteins were dominated by 11S globulin and 2S albumin, and the secondary structure was dominated by β-folding, with -SH and S-S varying greatly among different varieties. Sunflower meal proteins vary widely in terms of functional properties among different varieties, and specialized quality screening was necessary. This study provided a reference and theoretical support for understanding sunflower seeds to further promote the processing and utilization of sunflower seeds.

## 1. Introduction

Sunflower seeds are the seeds of plants in the Helianthus genus (*Helianthus annuus L.*) and one of the four most widely grown and consumed oil crops in the world [1,2], and their production had increased year by year to more than 47 million tons by 2023 (USDA) [3]. Sunflower seeds, composed of rind (shell), seed coat, cotyledon, and embryo [4], are primarily bifurcated into two categories, namely edible sunflower seed [5] and oil sunflower seed [6]. The former is characterized by a larger size and thick, angular skin and is frequently utilized for direct consumption [7], baking, and frying to produce confectionery items [8]. The latter, characterized by thin, black, or dark purple in color shells and smaller seeds are primarily employed in producing sunflower seed oil [7,9]. Sunflower seed oil is widely acclaimed in several countries in Europe, Mexico, and South America [10] compared to other vegetable oils owing to its easy availability and several health benefits (including less serum cholesterol [11], low-density lipoprotein levels, antioxidants, regulating blood pressure [12], anti-inflammatory, skin protection, and pain relief [13]. Sunflower seed oil is characterized by a relatively high proportion of unsaturated fatty acids, especially linoleic acid and oleic acid, conferring high nutritional value [14]. Furthermore, the residual meal from sunflower seeds after oil extraction contains rich protein (40~50%), with a high nutritional value and balanced amino acid composition, thus rendering it a high-quality plant protein resource [15].

Sunflower seed varieties are rich in resources; however, sunflowers are all mixed and harvested without scientific classification according to their uses, lacking special varieties and low product quality and efficiency, which seriously restricts the high-quality development of the sunflower seed processing industry. In recent years, much progress has been made in sunflower seed-related research, mainly focusing on sunflower seed [16], oil [17], and protein [18]. The raw material properties, physicochemical properties, and processing characteristics of sunflower seeds need to be further studied, but relevant reports are limited. In the context of industrial applications, conducting or grading screening of the raw materials is often imperative. The dimensions (length, width, height, and diameter), sphericity, fruit/kernel weight, hardness, brittleness, and other raw material indicators of sunflower seeds hold significant relevance for their screening during the processing. In addition to differences in raw material characteristics, the fatty acid composition of sunflower oil varies greatly among different varieties, and there are fewer related studies on it. Furthermore, there are fewer studies on the amino acid composition and functional properties of sunflower meal proteins from different varieties. It is of great significance to clarify the fatty acid, amino acid composition, and protein functional properties of sunflower seeds of different uses (edible and oil) and varieties for their application in different food fields. Therefore, it is necessary and urgent to systematically carry out the quality analysis of different varieties of sunflower seed raw materials and their products to improve the poor quality of products and industrial efficiency.

This study aimed to comprehensively compare and analyze the raw material characteristics, basic physicochemical properties, and processing characteristics of eight sunflower seeds (four edible and four oil sunflower seeds) and the correlation between these parameters. The macroscopic and microscopic differences in the raw materials of different varieties of sunflower seeds, the fatty acid composition of sunflower seed oil, the amino acid composition of meal protein, and the basic structure and functional characteristics of meal protein were compared and discussed simultaneously [19]. A proper understanding of the differences and correlations between different uses and varieties of sunflower seeds can offer valuable insights for the application of sunflower seeds in raw material screening and food processing.

## 2. Materials and Methods

### 2.1. Materials and Reagents

Raw materials from 8 sunflower seed varieties, including edible sunflower seeds (KBK, F9, F10, and 601) and oil sunflower seeds (NLY1, NLY2, 562, and S06) were collected from Xinjiang Academy of Agricultural Reclamation Sciences and Bayannur City Market. The reagents comprising anhydrous ethanol, petroleum ether, copper sulfate, potassium sulfate, sodium hydroxide, isooctane, sulfuric acid, boric acid, acetonitrile, glacial acetic acid, acetone, methyl red, bromocresol green, methylene blue, hydrochloric acid, potassium hydroxide, acetone, and Nile red were received from a research laboratory.

### 2.2. Quality Determination of Raw Materials

#### 2.2.1. Appearance and Microstructure of Sunflower Seeds

A suitable quantity of sunflower seeds was neatly arranged, and the appearance was photographed and sampled using a camera (D7200, Nikon, Tokyo, Japan). The microstructures of sunflower seeds were examined using Scanning Electron Microscopy (SEM, Hitachi SU8010, Tokyo, Japan). The shell was carefully removed without causing any damage to the raw materials of sunflower seeds, and the method of Xiang et al. [20] was slightly modified. Then it was observed under SEM following gold spraying with a magnification of ×500. The microscopic morphology of the semi-thin section cells of the sunflower seeds was observed by Confocal Laser Scanning Microscopy (CLSM TCS-SP8, Leica, Germany) [21]. The sunflower seeds were cut to an appropriate size, then moistened in the plant fixing solution, and semi-thinly sliced using a microtome. Semi-thin slices of sunflower seeds were stained with 0.2% fluorescein isothiocyanate and 0.1% Nile red, respectively. After a 3 min staining period in the absence of light, the excess staining medium on the surface was rinsed off with ×1 phosphate buffer, and the structure of sunflower seed cells was observed with a magnification of the original field of view ×63.

#### 2.2.2. Sunflower Seeds Engineering Characteristics

**Figure 1:**
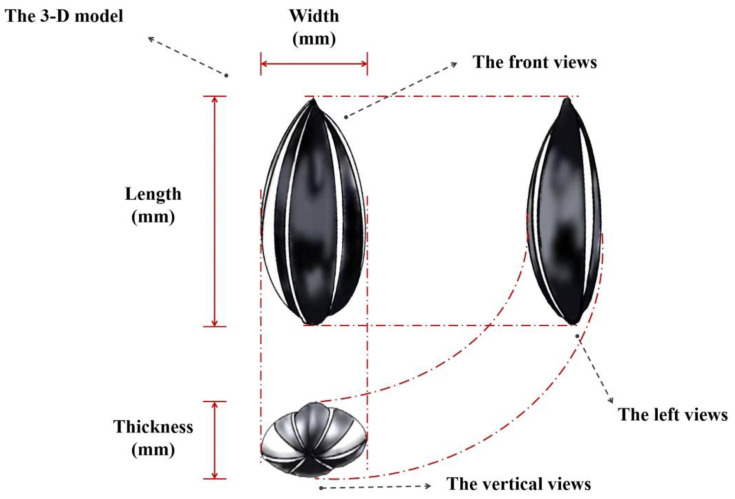
The 3D model of sunflower seeds.

A group of 100 sunflower seeds was randomly measured to determine their size, specifically length (L), width (W), and thickness (T), which were ascertained using a vernier caliper with a precision of ± 0.01 mm (the 3D model of sunflower seeds is shown in Figure 1). The diameter (D_r_), arithmetic mean diameter (D_α_), geometric mean diameter (D_g_), and sphericity (*ϕ*) of sunflower seeds were calculated, respectively. An analytical balance with a precision of ± 0.01 g was used to weigh 100 sunflower seeds, and the weight of these 100 seeds was recorded (W_hf_). The sunflower seeds were manually husked, and the weight of the kernel was measured (W_hk_). The hardness (H_d_), brittleness (B_t_), and cohesive force (C_h_) of the sunflower seed kernel were measured by TA-TX2i texture analyzer (Stable Micro Systems, London, UK). The probe type used was P/36R mm, the operation type was a return to start, and the pressure was measured under the strain mode. The waiting time was set at 0 s, and the test speed was maintained at 2.00 mm/s. Both the pre-test and post-test speeds were set at 2.00 mm/s; the speed after the test was 2.00 mm/s; and the compression degree (strain) was set at 40%.

(1) $$D_{r}=\frac{W+T}{2}$$

(2) $$D_{\alpha }=\frac{L+W+T}{3}$$

(3) $$D_{g}=\sqrt[{3}]{\mathrm{LWT}}$$

(4) $$\phi =\frac{\sqrt[{3}]{\mathrm{LWT}}}{L}$$

#### 2.2.3. Basic Composition of Sunflower Seeds

The moisture content was observed by drying the sample until a constant weight was achieved at 105 °C. The crude fat content of 2.0 g samples was determined by the Soxhlet extraction method, and the solvent was petroleum ether (boiling point 30~60 °C). The ash content of sunflower seed raw material was determined by heating the sample in a Muffle furnace at 550 °C until a constant weight was reached. The protein content of sunflower seeds was measured using the Kjeldahl nitrogen determination method, with a conversion coefficient of 5.3. The contents of moisture, ash, crude fat, and crude protein were determined according to the Chinese National Standard GB 5009.3-2016, GB 5009.4-2016, GB 5009.6-2016, and GB 5009.5-2016, respectively [22]. Petroleum ether (boiling point range 30–60 °C) was used to extract oil from different varieties of sunflower seeds at low temperatures; sunflower oil was obtained by rotary steaming, and the remaining sunflower seed meal was dried naturally in the fume hood for use. The dried sunflower seed meal was crushed through a 60-mesh sieve to obtain sunflower seed meal protein.

### 2.3. Analysis of the Fatty Acids of Sunflower Seed Oil

The sunflower oil was extracted through rotary steaming, and the residual sunflower seed meal was naturally dried in the fume hood for subsequent use. The oil samples were methylated using a methanolic KOH solution following the method described by Guo et al. [23,24] and then analyzed by a GC-2030 chromatograph (Shimadzu, Kyoto, Japan) [25]. The GC-2030 chromatograph is equipped with a CP-SIL 88 column (100 m × 0.25 μm × 0.2 mm; Supelco, Bellefonte, PA, USA) and a flame ionization detector (FID). The initial temperature of 60 °C was maintained for 5 min and then increased to 160 °C at a rate of 25 °C/min. After a time of 5 min at 160 °C, the temperature was again raised at a rate of 2 °C/min to achieve a final temperature of 225 °C. The sample was maintained at this final temperature for 15 min. The injection volume was 1 μL with a split ratio of 1:10, and helium (99.999%) was used as the carrier gas with a flow rate of 6.3 mL/min. The injector and interface temperatures were both 230 °C.

### 2.4. Amino Acid Composition of Sunflower Seed Meal Protein

The amino acid composition of sunflower seed meal protein was determined according to the method of Petraru et al. [26] using an automatic amino acid analyzer Aracus 300 (MembraPure GmBH, Berlin, GE, Germany).

### 2.5. SDS-PAGE of Sunflower Seed Meal Protein

The SDS-PAGE was conducted using the Mini-PROTEAN^®^ System (Bio-Rad, Hercules, CA, USA). The sunflower seed meal protein samples (2 mg) were dissolved in 0.5 mL of sample buffer (0.08 M Tris-HCl buffer, pH 6.8), 1% (*w*/*v*) SDS, 2% (*v*/*v*) 2-β-mercaptoethanol, 5% (*v*/*v*) glycerol, and 0.025% (*w*/*v*) bromophenol blue and mixed well [27]. The protein was subjected to SDS-PAGE analysis using a 5% concentrating gel and a 12% separating gel. A marker with a molecular weight range of 11~180 kDa (Sigma-Aldrich Co., St. Louis, MO, USA) was employed. 4 μL samples of supernatant were applied to the stacking gel slot and 1 L of electrode buffer was added. Electrophoresis was conducted initially at 30 V for 1.5 h, followed by a change to 60 V for an additional 2 h. After electrophoresis, the gel was stained with Coomassie brilliant blue R250. The decolorization was achieved using a methanol-ice acetic acid solution, and the photographic analysis was performed after decolorization. The images were finally taken using a Bio-Rad gel imaging system. Image Studio Lite (version 5.2, LI-COR Biosciences, Lincoln, NE, USA) The intensity of the stained bands is analyzed by Image Studio Lite to determine the relative ratio of specific proteins to the total protein content, providing an indication of the component purity of the sample.

### 2.6. FTIR of Sunflower Seed Meal Protein

The sunflower seed meal protein from various varieties was combined with KBr in a 1:100 ratio, ground with agate mortar, and pressed into transparent slices with a tablet press for detection. Fourier transform infrared (FTIR) spectroscopy was performed in a wavenumber range of 400~4000 cm^−1^ with 64 scans and a resolution of 2 cm^−1^. Within the amide, I band (1700~1600 cm^−1^), the relative proportions of the protein secondary structure were calculated using Fourier self-deconvolution, second derivative analysis, and curve fitting.

### 2.7. Free Sulfhydryl Groups and Disulfide Bonds of Sunflower Seed Meal Protein

The Ellman reagent was prepared first using Tris-glycine buffer (0.086 mol/L Tris, 0.09 mol/L glycine, 4 mmol/L EDTA, pH 8.0) and 4 mg DTNB reagent was added to 1 mL of Tris-glycine buffer. A slight modification was made to the method proposed by Chen and Zhang [28]. A 15 mg sample was dissolved in a 5 mL Tris-glycine −8 mol/L buffer solution, stirred for 30 min on a magnetic stirrer, and then centrifuged at 3000 r/min for 20 min. The supernatant was removed and 50 μL Ellman reagent was added, followed by a 1 h incubation at room temperature. The absorption at 412 nm (A412) was measured using a blank mixture without added protein, and the free sulfhydryl group content was calculated according to formula 5. For the total SH determination, a 4.7 g sample of guanidine hydrochloride protein was added to 15 mg of the sample, and the volume was adjusted to 5 mL with a Tris-glycine buffer (pH 8.0) containing 0.086 mol/L Tris, 0.09 mol/L, and 4 mmol/L EDTA. Then, 1 mL of sample solution was added to 4 mL of a solution containing 8 mol/L urea + 5 mol/L guanidine hydrochloride. After adding 0.1 mL of mercaptoethanol and allowing the reaction to proceed at room temperature for 1 h, 10 mL of a 12% trichloroacetic acid solution was added, followed by another 1 h reaction. The mixture was centrifuged at 5000 r/min for 10 min. The supernatant was discarded, and the precipitate was washed twice with 5 mL of a 12% trichloroacetic acid solution with each wash, followed by a 10 min centrifugation at 5000 r/min. The precipitate was dissolved in 10 mL of 8 mol/L urea, and 0.08 mL of a 4 mg/mL DTNB solution was added. Finally, 1 mL of the solution was taken and mixed with 5 mL of buffer solution. The absorbance was measured to compare color at 412 nm to obtain the absorption value.

(5) $$\mathrm{SH}(\mu \mathrm{mol}/g)=\frac{73.53A_{412}}{C}$$

(6) $$\mathrm{SS}(\mu \mathrm{mol}/g)=\frac{N_{2}-N_{1}}{2}$$

A412 represents absorbance, and C represents sample protein concentration (mg/mL). N1 represents the total free sulfhydryl, and N2 represents the total sulfhydryl content.

### 2.8. Functional Properties of Sunflower Seed Meal Protein

#### 2.8.1. Solubility

Solubility was measured according to the Li et al. [29] method; the sunflower seed protein samples of various varieties were prepared by accurately weighing 2 g of each sample and dispersing them in 90 mL of deionized water. The pH value was adjusted to 7.0 using either 1 M HCl or 1 M NaOH. The samples were stirred at room temperature for 2 h and subsequently centrifuged at 4000 r/min for 15 min. The protein content of the supernatant was determined using the Kjeldahl method (the conversion factor is 5.3). The nitrogen solubility index (NSI) of sunflower seed protein was calculated as the percentage of protein content in the supernatant relative to the total protein content in the sample.

#### 2.8.2. Foaming Property

A precise quantity of 0.5 g of sunflower seed meal protein was weighed and combined with 50 mL of deionized water, with the initial liquid height duly noted. Following this, the foam height was measured every 15 min by stirring at 12,000 r/min using a high-speed disperser for 2 min. The foamability (FC) was quantified as the ratio of the volume of the foam solution after shearing to the original volume. The foam stability (FS) was calculated as the ratio of the difference between the volume of the protein solution and the original volume after a 15 min standing period. The formulas were as follows:

(7) $$\mathrm{FC}(\%)=\frac{V_{1}\;-\;V_{0}}{V_{0}}$$

(8) $$\mathrm{FS}(\%)=\frac{V_{2}\;-\;V_{0}}{V_{0}}$$

V0 and V1 represent the volume before and after shearing, and V2 represents the volume after 15 min.

#### 2.8.3. Emulsifying Properties

A protein sample solution was prepared at a concentration of 1% using distilled water. Subsequently, 15 mL of protein solution was mixed with 5 mL of soybean oil in a 3:1 ratio, homogenized for 2 min at 12,000 r/min. At 0 and 10 min, 50 μL of emulsion was extracted from the bottom of the container and mixed with 5 mL (0.1%, *w*/*v*) SDS solution. The absorbance was measured at 500 nm wavelength at both 0 min (A_0_) and 10 min (A_10_) intervals using an ultraviolet spectrophotometer. The emulsification index (EAI) and emulsification stability index (ESI) were calculated using the following formula:

(9) $$\mathrm{EAI}=2\times T\frac{A_{0}\times N}{\varphi \times L\times C\times 10,000}$$

(10) $$\mathrm{ESI}=\frac{A_{0}\times \text{∆}T}{A_{0}\;-A_{10}}$$

In the above formula, T = 2.303, N represents the emulsion dilution factor, φ represents the oil volume fraction of the emulsion, C represents the sample concentration, mg/mL, L represents the optical path (set to 0.01), ΔT = 10 min.

#### 2.8.4. Water and Oil Holding Capacity

A precise quantity of 0.2 g sunflower seed meal protein was weighed and placed into a 10 mL centrifuge tube, to which 5 mL of water (oil) was added. The mixture was shaken for 2 min, allowed to stand for 5 min, and centrifuged at 9000 r/min for 15 min. The supernatant was removed, and the test tube was weighed. The water (oil) absorption capacity of the protein was determined as the amount of water (oil) bound per 100 g of sunflower protein.

### 2.9. Statistical Analysis

All data were processed and plotted by Origin 2022 software, and ANOVA analysis was performed by SPSS 16.0 software (SPSS Inc. Chicago, IL, USA). Furthermore, Origin 2022 software was used to perform a correlation analysis on raw material size characteristics, basic composition, processing characteristics, and basic physicochemical indices of different sunflower seeds. The significance of the differences between the means were evaluated by Duncan’s multiple range test with a confidence interval set at 95%.

## 3. Results and Discussion

### 3.1. Raw Material Quality Analysis

#### 3.1.1. Appearance and Microstructure of Sunflower Seeds

**Figure 2:**
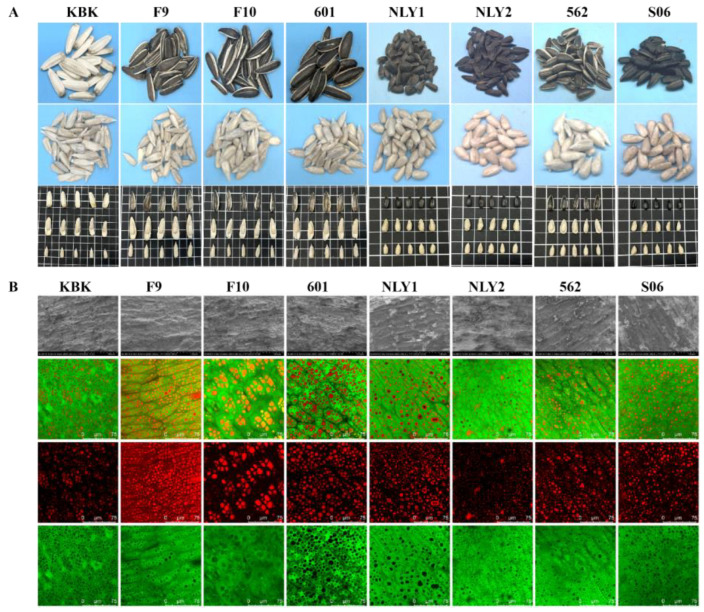
Appearance and microstructure of sunflower seeds for different uses and varieties. (**A**) appearance of sunflower seed; (**B**) microstructure of sunflower seed.

Both edible and oil sunflower seeds exhibited flat and elongated seed morphologies [30]. However, significant differences existed in the morphological characteristics, sensory characteristics, and microstructure of sunflower seeds for different usages (edible and oil) [6] and varieties (Figure 2). Edible sunflower seeds were flattened, large kernels with more than 2 cm in length as shown in Figure 2A. They were characterized by white and brown stripes, or a white-yellow shell, with a lighter luster. There was a large cavity between the kernel and the shell of edible sunflower seeds, and the flavor of spices was easily dispersed into this cavity during the baking process, thereby increasing the flavor of sunflower seeds, which provides the best physical basis for eating sunflower seeds as fried candy. The oil sunflower seeds were smaller, approximately 1 cm in length, and primarily dark brown or black with obvious luster. The seeds of the sunflower oil were tightly fitted to the shell, which facilitated the oil extraction during the pressing process.

The microstructure of sunflower seeds, as shown in Figure 2B, was characterized by oval-shaped long, tightly arranged between the cells with a minimal intracellular gap. The cell size, shape, and distribution of oil and protein varied significantly among different sunflower seed varieties. SEM revealed that the cells of edible sunflower seed cells are large, with minimal oil spillage in the cross-section. Protein bodies and starch granules are clearly visible in the cells, appearing as full and rounded globules with a dense globule distribution. The cells of oil sunflower seeds were slightly smaller. Due to their high-fat content, the cross-section of oil sunflower seeds was fully covered with oil, showing a large, bright oil halo in the SEM image. In the CLSM images, the long oval cells of sunflower seeds are clearly visible. The cell size of edible sunflower seed was 70–80 μm, while that of oil sunflower seed was slightly smaller, ranging from 30–60 μm. Furthermore, the oil in the cells of edible sunflower seed was relatively aggregated, existing as larger oil droplets within the cell matrix, and the protein distribution was larger. The oil in the cells of oil sunflower seed was more dispersed, appearing as small and numerous droplets, more uniformly dispersed in sunflower seed cells. The protein distribution in oil sunflower seed cells was more dispersed than in edible sunflower seed cells, which also confirmed that the protein content of oil sunflower seed was lower than that of edible sunflower seed.

#### 3.1.2. Sunflower Seeds Engineering Characteristics

The size characteristics and quality of sunflower seeds were of great significance for the harvest, cleaning, and grading of sunflower seeds in the whole processing chain [8]. Table 1 shows the triaxial size, size proportion, diameter, sphericity, hundred fruit weight, hundred kernel weight, and texture characteristics of sunflower seeds for different uses and varieties. Sunflower seeds were elongated seeds (length > width > thickness). The length, width, and thickness of 8 kinds of sunflower seeds were 10.23 ± 0.59~24.57 ± 1.19, 4.70 ± 0.2~9.36 ± 0.51, and 3.05 ± 0.24~4.89 ± 0.66 mm, respectively. The triaxial size of edible sunflower seeds was twice as large as that of oil sunflower seeds. There were significant differences in the size of sunflower seeds for different purposes and varieties. A comparison of edible and oil sunflower seeds revealed no significant differences in seed thicknesses. Therefore, sunflower seeds could be considered for grading and screening by length or width during the cleaning and grading process. Seeds with a lower aspect ratio were fuller at the same time. The aspect ratio of 8 sunflower seeds ranged from 1.81 ± 0.10~3.09 ± 0.20 mm, with edible sunflower seeds higher (2.62 ± 0.14~3.09 ± 0.20 mm) than that of oil sunflower seeds (1.81 ± 0.10~2.24 ± 0.34 mm). The flat shape of edible sunflower seeds provided a prerequisite for easy hulling. Among oil sunflower seeds, NLY2 was the fullest, with an aspect ratio of 1.81 ± 0.10 mm, which was also consistent with its appearance. The size of oil sunflower seeds was smaller than that of edible sunflower seeds except *ϕ*. The smaller size of *ϕ* indicated that the seeds were more spherical, indicating that seeds were not easily rotated during treatment [9]. The size of sunflower seeds ranged from 0.50 ± 0.02 to 0.57 ± 0.02 mm, which was of significant reference value for the seed hopper design of seed hopper for sunflower seed quality classification and screening. In terms of weight, the hundred fruit weight and kernel weight (19.53 ± 0.19~27.02 ± 0.25 g, 8.47 ± 0.28~13.49 ± 0.42 g) of edible sunflower seed were higher than that of oil sunflower seed (5.69 ± 0.28~8.23 ± 0.18 g, 4.13 ± 0.11~6.19 ± 0.14 g). However, the larger cavity of the edible sunflower seed resulted in a lower yield (43.38 ± 1.02~49.89 ± 1.10%), which was much lower than that of the oil sunflower seed (63.97 ± 0.65~81.79 ± 0.25%). Among them, S06 exhibited the highest yield (81.79 ± 0.25%), and its appearance showed a small cavity.

In addition, the edible sunflower seeds texture showed great variation among different varieties, which might be due to the high protein and low-fat content of edible sunflower seeds. The hardness (14,444 ± 1618~20,366 ± 2310 g) and brittleness (8657 ± 1601~13,453 ± 997 g) of edible sunflower were several times greater than that of oil sunflower seed (5218 ± 745~7303 ± 1227 g, 2759 ± 473~4139 ± 764 g), which was consistent with the microstructure trend. The cell structure of the edible sunflower seed was larger than that of the oil sunflower seed, and the protein bodies and starch granules revealed full and round spherules. The spherules were densely distributed in the cells, which gave great hardness and brittleness to the edible sunflower seed. However, oil sunflower cells were small and had a high fat content. During the extrusion process, the grease overflow enhanced the lubricity of sunflower seeds, and the hardness and brittleness were lower. Therefore, edible sunflower seeds should be used as raw materials for roasting and eating, and oil sunflower can be considered for use in the fields of oil pressing and butter [31].

#### 3.1.3. Basic Composition of Sunflower Seed

There are great differences in the basic composition of sunflower seeds for different varieties, which also determines their application fields [32]. This study conducted a comparative analysis of 4 edible and 4 oil sunflower seeds were selected for comparative analysis. The fat content of oil sunflower seeds ranged from 51.9 to 59.4%, which was higher than that of edible sunflower seeds (41.21~46.76%). Notably, the fat content of NLY2 oil sunflower seeds reached as high as 59.4%. In contrast to the fat content, the protein content of oil sunflower seed (18.46~22.27%) was lower than that of edible sunflower seed (25.53~28.53%). Among the oil sunflower seeds, the protein content of 562 was high, reaching 22.27%. It can be considered the oil and protein variety of sunflower. The water content of edible sunflower seed (4.40~5.08%) was generally higher than that of oil sunflower seed (2.72~3.60%). The water content also provided a certain medium for the Maillard reaction [33] of edible sunflower in the roasting of oil and protein, which may be an influential factor for the pleasant aroma and suitability of edible sunflower after roasting. In addition, the water content level also affects the storage and freshness of sunflower seeds. More attention should be paid to the phenomenon of moisture and mildew in the storage process of sunflower, and different conditions can be considered for the storage of edible sunflower seed and oil sunflower seed. Ash is the mineral and inorganic salt and other impurities in the food after the burning oxidation of the remaining substance, to a certain extent can reflect the amount of mineral elements in the food [34]. The ash content of sunflower seeds of different varieties also varied greatly. Except for S06, the ash content of edible sunflower seeds was higher than that of edible sunflower seeds, and there were more non-volatile mineral elements in edible sunflower seeds, which may also contribute to the production of confectionery.

### 3.2. Fatty Acid Composition of Sunflower Seed Oil

Sunflower seeds primarily contain fatty acids such as oleic acid (C18:1), linoleic acid (C18:2), stearic acid (C18:0) and palmitic acid (C16:0), and so on [35]. Depending on the degree of unsaturation, the content of saturated fatty acids in sunflower seeds of different varieties ranged from 5.91 to 10.95 g/100 g, monounsaturated fatty acids from 18.94 ± 1.12 to 79.60 ± 2.58 g/100 g, and polyunsaturated fatty acids from 15.19 to 54.84 g/100 g. Sunflower oil is a vegetable oil dominated by unsaturated fatty acids (70.72 ± 1.57~94.79 ± 2.75). There were significant differences among different varieties, so it was necessary to carry out quality evaluation and special variety screening for different varieties of sunflower seeds. C18:1 and C18:2 are essential fatty acids for the human body and have been confirmed to have positive effects [36] on blood lipids [37], anti-inflammatory [38], and anti-atherosclerosis [39]. However, there are great differences among different sunflower seeds, including C18:1 (18.94 to 79.30 g/100 g), and C18:2 (10.11~51.72 g/100 g). KBK and S06 had O/L ≈ 1:1, 601, NLY1 and NLY2 had high C18:1 content; F9 and F10 had high C18:2 content, and the C18:1 content of NLY1 amounted to 79.30 ± 3.11 g/100 g, which is a high oleic acid variety. High oleic acid vegetable oil is considered to be a kind of vegetable oil beneficial to human health, so NLY1 was considered as a sunflower variety specialized for edible oil and fat processing [40]. Moreover, the ratio of oleic acid to linoleic acid (O/L) in oil has a decisive significance for its application. Wei et al.’s [40] study showed that O/L of 1:1 was suitable for frying oil. The ratio of oleic acid to linoleic acid in different varieties of sunflower seed oil was 1:0.36~7.84, of which the oleic acid of S06 was ≈1:1, which was suitable for the special processing variety of frying oil from the perspective of fatty acid composition. The significant differences in the fatty acid composition of sunflower seeds may be related to their varietal differences [41], high oleic acid, high linoleic acid, and high stearic acid varieties that have a greater impact on the overall fatty acid composition.

### 3.3. Amino Acid Composition Analysis of Sunflower Seed Meal Protein

**Figure 3:**
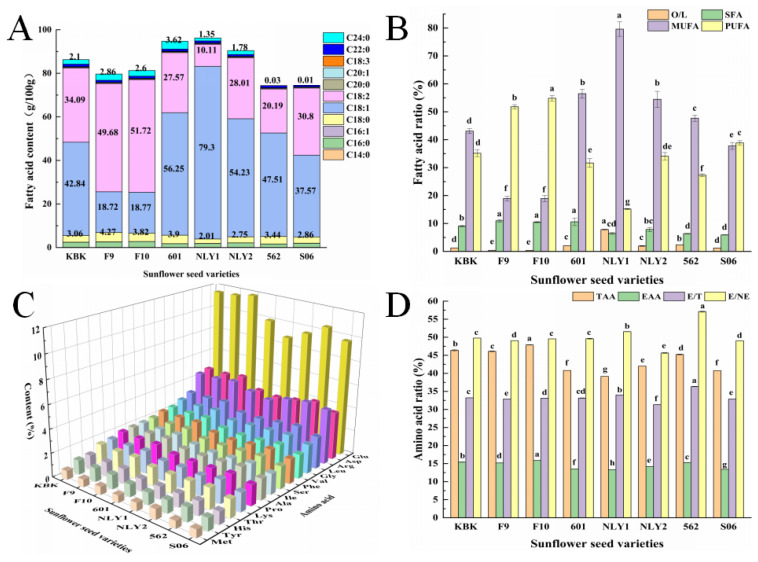
Fatty acid and amino acid composition of sunflower seed oil with different uses and varieties. (**A**) fatty acid; (**B**) fatty acid ratio; (**C**) amino acid; (**D**) amino acid ratio. Note: Different superscript letters indicate significant differences (*p* < 0.05).

It can be observed from Figure 3C that the amino acid composition of sunflower seed meal protein of all varieties has the highest Glu content (8.884~11.855 g/100 g), followed by Asp (3.921~4.864 g/100 g) and Arg (4.0265~4.7995 g/100 g). Glu causes sweet, salty, sour, and bitter flavors and is often used as a refreshing substance or raw material for monosodium glutamate [42]. The proportion of Glu in the amino acid composition of sunflower seed meal protein reaches about 25%, and the preparation of fresh flavor peptides with it has a greater advantage [43]. Evaluation of the nutritional value of protein should not only look at its content, but also examine its quality, and the quality of protein depends mainly on its amino acid composition and its proportionality. The total amino acid content of different varieties of sunflower seed meal protein accounted for 39.16~47.89 g/100 g, of which the essential amino acid (EAA) content accounted for 13.32~15.87 g/100 g, and the ratio of EAA to total amino acids ranged from 31.34 to 34.01, which has a better nutritional value, but there are significant differences between varieties. In the oil sunflower seed protein, except for NLY2, which had a higher content of EAA (15.27 g/100 g), the EAA content of other varieties of oil sunflower seed meal protein was smaller than that of edible sunflower seed meal protein (13.52~25.87 g/100 g). This indicates that the protein nutritional value of edible sunflower seeds may be slightly higher than that of oil sunflower seeds.

### 3.4. Correlation Analysis between Raw Materials and Processing Characteristics

**Figure 4:**
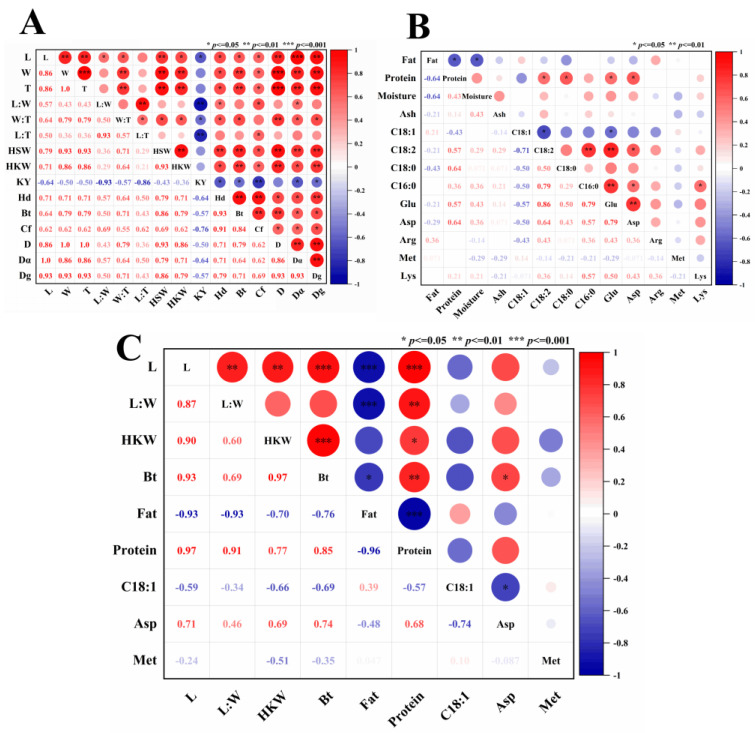
Correlation between different uses and varieties of sunflower seed raw materials, basic physicochemical and processing characteristics. (**A**) raw materials; (**B**) basic physicochemical; (**C**) processing characteristics.

A Pearson correlation analysis was performed on 28 quality indicators, including raw materials, and processing characteristics of edible and oil sunflower seeds (Figure 4). The selection of raw material indicators for sunflower seeds included length, aspect ratio, diameter, arithmetic mean diameter, geometric mean diameter, hundred fruit and kernel weight, and their texture characteristics. All other indicators showed a significant positive correlation except for the aspect ratio in the raw material indicators. Among them, length was significantly positively correlated with diameter (R = 0.86, *p* < 0.01), geometric mean diameter (R = 0.93, *p* < 0.01), and hundred fruit weight (R = 0.79, *p* < 0.01). The diameter was significantly positively correlated with the weight of a hundred fruits (R = 0.93, *p* < 0.01), the weight of a hundred kernels (R = 0.86, *p* < 0.01), hardness (R = 0.71, *p* < 0.05), and brittleness (R = 0.79, *p* < 0.01). Hardness and brittleness were also important indicators affecting the processing of sunflower seed raw materials. The hardness and brittleness of sunflower seeds were significantly positively correlated with each indicator (R > 0.50, *p* < 0.05).

The fat content of sunflower seeds was significantly negatively correlated with protein content (R = −0.64, *p* < 0.05) and moisture content (R = −0.64, *p* < 0.05) in the physicochemical and processing indicators of sunflower seeds, while the correlation with ash content was poor. The C18:1 showed a significant negative correlation with other indicators (R < −0.43, *p* < 0.05) among the processing characteristics of different varieties of sunflower seeds, C18:1 showed a significant negative correlation with other indicators (R < −0.43, *p* < 0.05). There was a significant positive correlation between C18:2 and C16:0 (R = 0.79, *p* < 0.01), Glu (R = 0.86, *p* < 0.01), and Asp (R = 0.64, *p* < 0.01). Met was positively correlated with C18:1 and Lys but negatively correlated with other indicators. This was due to the reason that significant differences were observed in fatty acid composition among different varieties of sunflower seeds, including those with high oleic acid, high linoleic acid, and high stearic acid content [35,40]. Therefore, it was essential to important to screen specialized varieties for different varieties of sunflower seeds.

The results indicated that there was a significant correlation between the raw material indicators of different varieties of sunflower seeds and their physical, chemical, and processing indicators. A single indicator can influence or facilitate changes in several other indicators. Therefore, the trend of a single indicator can to some extent represent the trend of several indicators to some extent. In subsequent analysis, indicators with the same trend can be reduced. The 15 sunflower seed raw material indicators could be represented by four indicators such as L, L/W, HKW, and Bt. The 13 physicochemical and processing indicators can be represented by 5 indicators, including Fat, Pro, C18:1, Asp, and Met.

The correlation analysis of 9 screened indicators revealed that L was negatively correlated with Fat (R = −0.93, *p* < 0.001), C18:1 (R = −0.59, *p* < 0.05), Met (R = −0.24, *p* < 0.05), and significantly positively correlated with L:W (R = 0.87, *p* < 0.01), HKW (R = 0.90, *p* < 0.01), and Bt (R = 0.93, *p* < 0.001). Protein was negatively correlated with Fat (R = −0.96, *p* < 0.001) and C18:1 (R = −0.57, *p* < 0.05), and positively correlated with other indicators. C18:1 was positively correlated with Fat (R = 0.39, *p* < 0.05). Asp showed a significant negative correlation with Fat (R = −0.48, *p* < 0.05), C18:1 (R = −0.74, *p* < 0.05), and a significant positive correlation with other indicators. By combining the analysis of raw materials, physicochemical properties, and processing characteristics, the 9 indicators were reduced to 4 indicators, namely L, Protein, C18:1, and Asp.

### 3.5. SDS-PAGE Analysis of Sunflower Seed Protein Powder

**Figure 5:**
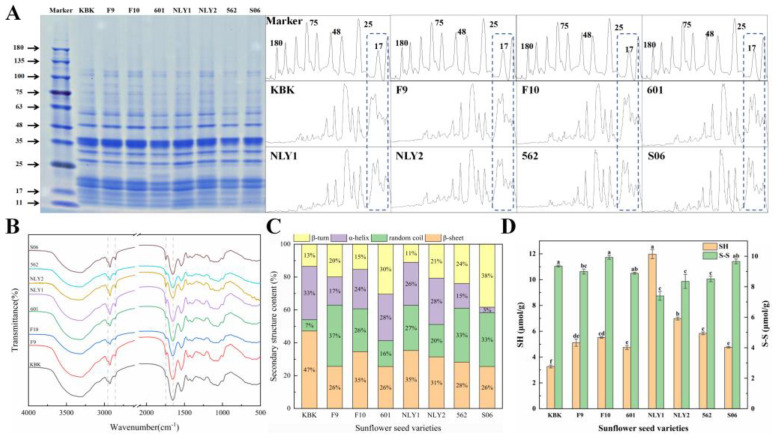
The basic structure of protein in sunflower seed meal with different uses and varieties. (**A**) SDS-PAGE; (**B**) FTIR; (**C**) secondary structure; (**D**) -SH and S-S. Note: Different superscript letters indicate significant differences (*p* < 0.05).

The overall distribution of protein bands in different varieties of sunflower meal remained consistent, mainly small molecular weight proteins with subunit contents below 120 KDa. It comprised 10 main bands with relative molecular weight distribution in the range of 11–63 KDa, aligning with the range reported by Jiang et al. [44]. In the electrophoretic diagram, the 11–20 KDa range primarily consisted of sunflower seed 2S albumin, accounting for 28.8–30.0% of the total, while the 26–63 KDa range was mainly sunflower seed 11S globulin, accounting for 45.3–45.9% of the total. There were differences in protein composition among different varieties of sunflower seeds. Studies have shown that 11S, 2S, and 11S/2S were closely associated with functional properties [45]. Sunflower seed 2S albumin was more soluble than 11S globulin, and higher levels of 2S contributed more to the increase in solubility. In subsequent studies, it may be worth considering adjusting the proportion of protein components to enhance the functional properties of sunflower seed protein [46]. In addition to 11S and 2S, other bands can be observed in Figure 5A, indicating the presence of other protein components other than 11S and 2S in sunflower seed meal protein [47].

### 3.6. FTIR Analysis of Sunflower Seed Protein Powder

The characteristic absorption of molecules can be characterized by wave number, location, peak number, and intensity of peaks in the FTIR test [48]. The infrared spectra of different sunflower seed varieties were generally consistent as shown in Figure 5B, indicating that the functional groups in the structure of different sunflower seed varieties and the molecular characteristics of sunflower meal proteins were consistent. Among them, 3500–3000 cm^−1^ was attributed to the wide peak caused by the stretching vibration of the free O-H bond and the amino N-H bond [49], and the KBK absorption peak was larger here. At 2931 cm^−1^ and 2857 cm^−1^, the absorption peak of oil sunflower at 2857 cm^−1^ was slightly stronger than that of edible sunflower seed. The peaks observed near 1662, 1540, and 1240 cm^−1^ were attributed to the -C=O stretching vibration (amido band I), the N-H bond bending vibration (amido band II), and the C-N stretching and N-H bending vibration (amido band III) [50], respectively. At 1720 cm^−1^, the absorption peak was attributed to the ester bond [51] inside sunflower seed meal protein, and the absorption intensity of oil sunflower was greater than that of edible sunflower. The fitting secondary structure of sunflower seed meal protein is shown in Figure 5C. The protein secondary structure of different varieties of sunflower meal was significantly different. The secondary protein structure of sunflower meal was mainly *β*-sheet, accounting for 25.56–47.15%, and the highest content of KBK was 47.15%. The *α*-helix content varied greatly, ranging from 3.39–32.52%, *β*-turn content from 11.04–38.32%, and random coil content from 6.87–35.30%. The protein secondary structure of different sunflower seed meal varieties varied greatly, which led to great differences in their functional properties.

### 3.7. Analysis of Free Sulfhydryl and Disulfide Bonds

Disulfide bonds play a crucial role in building protein structures and maintaining their functional properties [52]. The oxidation of two sulfhydryl groups in the protein formed disulfide bonds, which are covalent bonds. Conversely, disulfide bonds can also be reduced to sulfhydryl groups, but the structure of the protein will become loose after forming sulfhydryl groups. The contents of the free sulfhydryl group [53] and disulfide bonds in different sunflower seed meal varieties were 3.27–11.97 and 7.39–9.92 μmol/g, respectively as shown in Figure 5D. The content of the free sulfhydryl group (3.27–5.51 μmol/g) in edible sunflower seed meal protein was lower than that in oil sunflower seed (4.77–11.97 μmol/g). There were significant differences in the secondary structure of sunflower meal protein between different applications and different varieties, which was necessary for screening special varieties for processing. It was observed that a higher content of the disulfide bond demonstrated a greater degree of intermolecular aggregation, which led to a decrease in protein solubility [52]. In NLY1, the content of the free sulfhydryl group in NLY1 was the highest (11.97 μmol/g), and the content of the disulfide bond was the lowest (7.39 μmol/g), which also unveiled that NLY1 would have good solubility in all varieties.

### 3.8. Analysis of Functional Properties of Sunflower Seed Meal Protein

**Figure 6:**
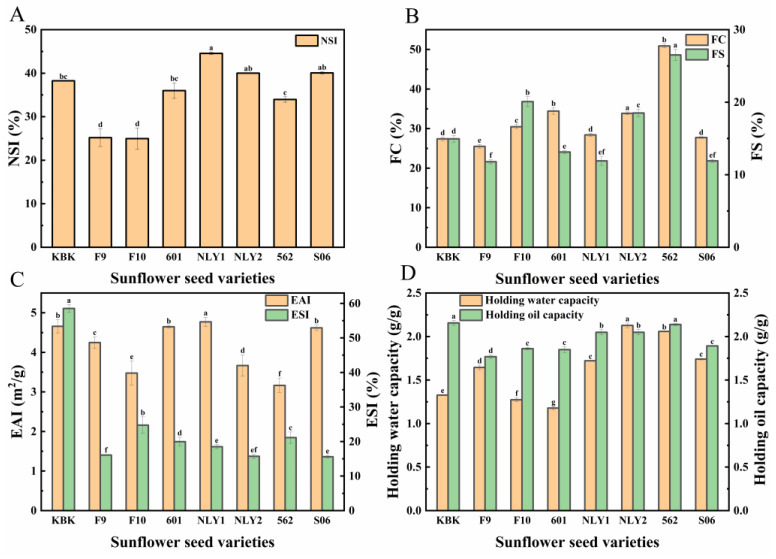
Functional characteristics of sunflower seed meal protein with different uses and varieties. (**A**) NSI; (**B**) foaming and foaming stability; (**C**) emulsification and emulsion stability; (**D**) holding water/oil capacity. Note: Different superscript letters indicate significant differences (*p* < 0.05).

The functional properties of proteins are pivotal factors that determine their application [54]. Solubility is one of the major quality criteria for the application of sunflower seed protein in food [27], and it is also a prerequisite for other functional properties, which affect the functional properties of sunflower seed protein such as foaming and emulsification [55]. The nitrogen solubility index (NSI) of different varieties of sunflower meal protein was represented in Figure 6A, which ranged from 24.97 to 44.56%. In addition, there were great differences among varieties, and oil sunflower seed was better than edible sunflower seed on the whole. The solubility of NLY1 was the highest, reaching 44.56%. As mentioned above, the 2S albumin of sunflower seeds contributed a lot to the solubility, and the content of 2S albumin of NLY1 accounted for 30.0%, which substantiated the good solubility of NLY1.

Foam property is one of the important functional properties in food processing technology [56], especially for some crisp food and foam drinks, mainly cakes, ice cream [57], and beer [58]. The foamability and foam stability of different sunflower meal proteins ranged from 25.5 to 50.9% and 11.79 to 20.08%, with significant differences among different sunflower meal proteins (Figure 6B). Among them, 562 showed good foaming properties (50.9%) and foaming stability (20.08%), and 562 varieties could be considered as foaming food raw materials.

Proteins can facilitate the mixing of oil and water, possessing the emulsification ability to maintain a stable mixture state of oil and water without separation [59]. In food, proteins can reduce the tension at the interface between water and oil, thereby preventing the accumulation of oil droplets and enhancing stability [60]. The emulsification properties of different sunflower seed meal proteins ranged from 3.16 to 4.75 m^2^/g, and the emulsification stability ranged from 15.54 to 58.35%, with significant differences among different varieties (Figure 6C). KBK exhibited superior comprehensive properties, with emulsification of 4.63 m^2^/g, and emulsification stability of 58.35%, which was better than other varieties of sunflower seed meal protein. The 11S protein content in KBK was at a high level, which had a positive effect on its emulsification and emulsification stability. KBK will have great application value in the fields of raw sauce, emulsion, cream and ice cream regardless of the fat content.

The water-holding capacity of protein, also known as hydration, and oil-holding capacity generally refer to the ability of protein to adsorb fat [61]. The water and oil holding capacity of different varieties of sunflower seed meal are represented in Figure 6D. The water holding capacity (1.17~2.11 *g*/*g*) of different sunflower seed meal protein had a maximum difference, while the oil holding capacity (1.78~2.14 *g*/*g*) had a minimum difference, and oil sunflower seed was better than edible sunflower seed on the whole. Among them, NLY2 and 562 had better water and oil holding capacity, with water and oil holding capacity reaching 2.11 *g*/*g* and 2.06 *g*/*g*, respectively, and oil holding capacity reaching 2.06 *g*/*g* and 2.13 *g*/*g*, respectively, which was better than soybean protein [62]. Water and oil holding capacity had a good effect on baking pastries, sauces, and meat products [63], so NLY2 and 562 can be considered for application in pastry and meat products and other fields.

## 4. Conclusions

In this study, raw material, physicochemical, and processing characteristics of eight sunflower varieties (four edible and four oil sunflower seeds) were comparatively analyzed. Significant differences existed between edible and oil sunflower seeds for each characteristic. With regard to the macro and micro structures of sunflower seed raw materials, the triaxial dimensions, hardness, brittleness, and cellular structure of edible sunflower seeds were larger than those of oil sunflower seeds. In the basic physicochemical composition, the fat content of oil sunflower seeds (51.9–59.4%) were higher than that of edible sunflower seeds (41.21–46.76%), while on the contrary, the protein content (18.46–22.27%) were smaller than that of edible sunflower (25.53–28.53%). Sunflower seed oil was mainly dominated by unsaturated fatty acids (66.89–89.79 g/100 g), with oleic acid and linoleic acid accounting for a relatively large proportion. Among them, the oleic acid content of NLY1 reaches 79.30 ± 3.11 g/100 g, which belongs to high oleic acid varieties and can be used as a special variety for oil processing. The ratio of essential amino acids to total amino acids in sunflower seed meal protein is 31.34~34.01, which has good nutritional value. Raw material, physicochemical, and processing characteristics correlation analysis initially screened out Length, Protein, C18:1, and Asp can be used as the main indicators of its quality evaluation. In addition, sunflower seed meal proteins were mainly dominated by 11S globulin, 2S albumin, and β- sheet, and -SH and S-S varied greatly among different varieties. Regarding functional properties, NLY1 had better solubility, 562 had better foaming (50.9%) and foaming stability (20.08%), and KBK had better emulsification (4.63 m2/g) and emulsion stability (58.35%). In summary, the characteristics of different varieties of sunflower seeds differ significantly from each other, and it is necessary and urgent to systematically carry out the quality analysis of different varieties of sunflower seeds raw materials and their products in order to improve the poor quality of the products and industrial efficiency.

## Figures and Tables

**Table 1:** Characteristics of sunflower seed raw materials with different uses and varieties.

Type	Edible Sunflower Seeds				Oil Sunflower Seeds			
	KBK	F9	F10	601	NLY1	NLY2	562	S06
Lee	24.33 ± 1.19 ^a^	24.57 ± 1.19 ^a^	24.39 ± 1.40 ^a^	21.88 ± 1.32 ^b^	11.30 ± 0.44 ^d^	11.41 ± 0.40 ^d^	12.65 ± 0.44 ^c^	10.23 ± 0.59 ^e^
W/mm	7.87 ± 0.58 ^b^	9.36 ± 0.51 ^a^	9.19 ± 0.83 ^a^	8.30 ± 0.73 ^b^	5.05 ± 0.38 ^e^	6.30 ± 0.34 ^c^	5.64 ± 0.39 ^d^	4.70 ± 0.28 ^e^
T/mm	4.12 ± 0.25 ^cd^	4.89 ± 0.66 ^a^	4.47 ± 0.45 ^b^	4.22 ± 0.53 ^bc^	3.43 ± 0.24 ^f^	3.80 ± 0.26 ^de^	3.59 ± 0.33 ^ef^	3.05 ± 0.24 ^g^
L/W	3.09 ± 0.20 ^a^	2.62 ± 0.14 ^b^	2.67 ± 0.24 ^b^	2.64 ± 0.24 ^b^	2.24 ± 0.17 ^c^	1.81 ± 0.10 ^d^	2.24 ± 0.34 ^c^	2.18 ± 0.20 ^c^
W/T	1.91 ± 0.13 ^b^	1.92 ± 0.25 ^ab^	2.07 ± 0.22 ^a^	1.97 ± 0.17 ^ab^	1.47 ± 0.14 ^d^	1.66 ± 0.11 ^c^	1.57 ± 0.13 ^cd^	1.54 ± 0.14 ^cd^
L/T	5.91 ± 0.47 ^a^	5.11 ± 0.64 ^c^	5.50 ± 0.54 ^b^	5.26 ± 0.65 ^bc^	3.29 ± 0.30 ^de^	3.00 ± 0.25 ^e^	3.52 ± 0.34 ^d^	3.35 ± 0.38 ^de^
D_r_/mm	5.99 ± 0.37 ^b^	7.12 ± 0.50 ^a^	6.83 ± 0.55 ^a^	6.26 ± 0.59 ^b^	4.24 ± 0.31 ^e^	5.05 ± 0.25 ^c^	4.61 ± 0.32 ^d^	3.88 ± 0.20 ^f^
D_α_/mm	12.11 ± 0.54 ^b^	12.94 ± 0.62 ^a^	12.68 ± 0.66 ^a^	11.46 ± 0.70 ^c^	6.59 ± 0.35 ^e^	7.17 ± 0.23 ^d^	7.29 ± 0.33 ^d^	6.00 ± 0.20 ^f^
D_g_/mm	9.23 ± 0.43 ^b^	10.38 ± 0.67 ^a^	9.99 ± 0.62 ^a^	9.13 ± 0.71 ^b^	5.81 ± 0.34 ^d^	6.48 ± 0.24 ^c^	6.34 ± 0.36 ^c^	5.27 ± 0.18 ^e^
*ϕ*	0.38 ± 0.02 ^d^	0.42 ± 0.02 ^c^	0.41 ± 0.02 ^c^	0.42 ± 0.03 ^c^	0.51 ± 0.77 ^b^	0.57 ± 0.02 ^a^	0.50 ± 0.02 ^b^	0.52 ± 0.03 ^b^
W_hf_/g	19.53 ± 0.19 ^c^	27.02 ± 0.25 ^a^	24.29 ± 0.34 ^b^	19.81 ± 0.14 ^c^	7.46 ± 0.11 ^e^	8.23 ± 0.18 ^d^	6.45 ± 0.11 ^f^	5.69 ± 0.28 ^g^
W_hk_/g	8.47 ± 0.28 ^d^	13.49 ± 0.42 ^a^	11.31 ± 0.25 ^b^	9.68 ± 0.12 ^c^	5.52 ± 0.04 ^f^	6.19 ± 0.14 ^e^	4.13 ± 0.11 ^g^	4.65 ± 0.21 ^g^
KY	43.38 ± 1.02 ^f^	49.89 ± 1.10 ^d^	46.58 ± 0.54 ^e^	48.86 ± 0.26 ^d^	73.96 ± 0.62 ^b^	74.62 ± 0.70 ^b^	63.97 ± 0.65 ^c^	81.79 ± 0.25 ^a^
H_d_/g	15,179 ± 3285 ^c^	18,186 ± 2291 ^b^	20,366 ± 2309 ^a^	14,444 ± 1618 ^c^	7303 ± 1227 ^d^	6896 ± 1103 ^d^	6882 ± 832 ^d^	5218 ± 745 ^e^
B_t_/g	8657 ± 1600 ^b^	13,333 ± 318	13,453 ± 997 ^a^	8809 ± 1170 ^b^	4139 ± 764 ^c^	3857 ± 734 ^cd^	3684 ± 505 ^cd^	2759 ± 473 ^d^
C_h_	0.69 ± 0.07 ^a^	0.69 ± 0.07 ^a^	0.69 ± 0.06 ^a^	0.57 ± 0.06 ^b^	0.48 ± 0.07 ^c^	0.42 ± 0.06 ^d^	0.33 ± 0.03 ^e^	0.28 ± 0.03 ^f^
Crude fat/%	41.21 ± 0.41 ^e^	45.24 ± 0.09 ^cd^	46.76 ± 0.09 ^c^	42.69 ± 0.64 ^de^	56.97 ± 1.30 ^a^	59.40 ± 1.33 ^a^	51.90 ± 0.46 ^b^	58.05 ± 0.41 ^a^
Crude protein/%	28.53 ± 0.08 ^a^	26.82 ± 0.18 ^a^	26.96 ± 0.77 ^b^	25.53 ± 0.14 ^b^	18.46 ± 0.14 ^d^	18.54 ± 0.27 ^d^	22.27 ± 0.73 ^c^	18.56 ± 0.33 ^d^
Moisture/%	5.08 ± 0.12 ^a^	4.40 ± 0.26 ^b^	4.41 ± 0.19 ^b^	4.49 ± 0.22 ^ab^	3.60 ± 0.31 ^c^	3.26 ± 0.09 ^cd^	2.72 ± 0.37 ^d^	3.21 ± 0.28 ^cd^
Ash/%	3.62 ± 0.07 ^ab^	3.52 ± 0.10 ^ab^	3.70 ± 0.06 ^a^	3.71 ± 0.13 ^a^	3.20 ± 0.03 ^bc^	2.96 ± 0.04 ^cd^	2.74 ± 0.24 ^d^	3.85 ± 0.15 ^a^

Note: Different superscript letters indicate significant differences (*p* < 0.05).

## Data Availability

The original contributions presented in the study are included in the article, further inquiries can be directed to the corresponding author.
